# Sr_9_La_2_(WO_6_)_4_ containing [WO_6_] octa­hedra

**DOI:** 10.1107/S2056989022006648

**Published:** 2022-07-05

**Authors:** Rayko Simura, Tomoki Watanabe, Hisanori Yamane

**Affiliations:** a Institute of Multidisciplinary Research for Advanced Materials, Tohoku, University, 2-1-1 Katahira, Aoba-ku, Sendai, 980-8577, Japan; Vienna University of Technology, Austria

**Keywords:** crystal structure, tungstate, double perovskite

## Abstract

A polycrystalline sample of Sr_9_La_2_(WO_6_)_4_, nona­strontium dilanthanum tetra­kis­[orthotungstate(VI)], was prepared by heating a compacted powder mixture of SrCO_3_, WO_3_, and La_2_O_3_ with an Sr:La:W molar ratio of 9:2:4 at 1473 K. X-ray crystal structure analysis was performed for a Sr_9_La_2_(WO_6_)_4_ single-crystal grain grown by reheating the sample at 1673 K. Sr_9_La_2_(WO_6_)_4_ crystallizes with four formula units in the tetra­gonal space group *I*4_1_/*a* and is isotypic with Sr_11_(ReO_6_)_4_. Two W sites with site symmetries of  are located at the center of isolated [WO_6_] octa­hedra, and four mixed (Sr/La) sites are surrounded by eight to twelve O atoms of the [WO_6_] octa­hedra. The structure of Sr_9_La_2_(WO_6_)_4_ can be described on the basis of the double-perovskite structure with [WO_6_] and [(Sr/La)O_*x*_] polyhedra alternately placed, and a vacancy (□).

## Abstract 2

The crystal structure of Sr_9_La_2_(WO_6_)_4_ can be derived on the basis of the double-perovskite structure type. [WO_6_] octa­hedra and [(Sr/La)O_*x*_] polyhedra are alternately placed in a distorted simple lattice with an additional vacancy (□).

## 1. Chemical context

The alkaline-earth (*A*) rare-earth (*Ln*) tungstates *A*_9_ *Ln*_2_(WO_6_)_4_ have attracted attention as host crystals of phosphors, and various luminescence properties of these tungstates doped with activators such as Eu^3+^ and Mn^4+^ have been evaluated. For example, emissions of Eu^3+^ at ∼615 nm excited by ∼395 nm wavelength light have been reported for Sr_9_Gd_1.5_Eu_0.5_(WO_6_)_4_ (Blasse & Kemmler-Sack, 1983 ▸), Ca_9_Gd_2–*x*_Eu_*x*_(WO_6_)_4_ (Zeng *et al.*, 2013 ▸), Ca_9_Eu_2_(WO_6_)_4_ (Qin *et al.*, 2012 ▸; Zeng *et al.*, 2010 ▸), Sr_9_Eu_2_(WO_6_)_4_ (Qin *et al.*, 2012 ▸; Blasse & Kemmler-Sack, 1983 ▸; Zeng *et al.*, 2010 ▸), and Ca_9–*x*_Sr_*x*_Eu_2_(WO_6_)_4_ (Zeng *et al.*, 2009 ▸). Mn^4+^-doped Sr_9_Y_2_(WO_6_)_4_ (Shi *et al.*, 2019 ▸) and Mn^4+^/Mg^2+^-doped Sr_9_Y_2_(WO_6_)_4_ (Zhou *et al.*, 2020 ▸) were also studied, and deep-red luminescence with broad emission maxima at ∼680 nm were observed under excitation by light with a wavelength of 365 nm.

Unit-cell parameters of a tetra­gonal cell with *a* = 11.664 (2) Å, *c* = 16.335 (4) Å (Smirnov *et al.*, 1987 ▸) and *a* = 16.44 (7) Å, *c* = 16.32 (3) Å (Kemmler-Sack & Ehmann, 1981 ▸) have been reported for Sr_9_La_2_(WO_6_)_4_. However, details of the crystal structure, including atom positions, have not been clarified up to now. Sr_9_ *Ln*_2_(WO_6_)_4_ compounds prepared by substituting *Ln* (a rare-earth element) for La in Sr_9_La_2_(WO_6_)_4_ have also been reported. These materials have tetra­gonal symmetry for *Ln* = La, Pr, and Nd; cubic (high-temperature phase) and tetra­gonal (low-temperature phase) symmetry for Sm, Eu, and Gd; monoclinic symmetry for Tb and Dy; and cubic symmetry for Ho, Er, Tm, and Y (Kemmler-Sack & Ehmann, 1981 ▸). The Sr atoms of Sr_9_La_2_(WO_6_)_4_ can also be replaced with Ca or Ba. For Ca_9_ *Ln*_2_(WO_6_)_4_ (*Ln* = Nd, Sm, Eu, Gd, Tb, Dy), lattice parameters of a tetra­gonal unit-cell with 11.05 ≤ *a* ≤ 11.13 Å and 16.37 ≤ *c* ≤ 16.42 Å and space group *I*4_1_/*a* have been reported (Smirnov *et al.*, 1987 ▸). Ba_9_ *Ln*_2_(WO_6_)_4_ compounds (*Ln* = La, Nd, Sm, Eu) are cubic (8.50 ≤ *a* ≤ 8.56 Å; Betz *et al.*, 1982 ▸). The crystal structures of Sr_9_Gd_2_(WO_6_)_4_ [*Fm* 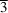 , *a* = 16.47013 (6) Å] and Ba_9_La_2_(WO_6_)_4_ [*Fm* 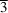 , *a* = 17.12339 (15) Å] have been fully analyzed (Ijdo *et al.*, 2016 ▸). However, atomic positions for the tetra­gonal structures of Ca_9_ *Ln*_2_(WO_6_)_4_ (*Ln* = Nd, Sm, Eu, Gd, Tb, Dy) compounds have not been determined.

Here, we report on synthesis and crystal structure analysis of Sr_9_La_2_(WO_6_)_4_.

## 2. Structural commentary

**Figure 1:**
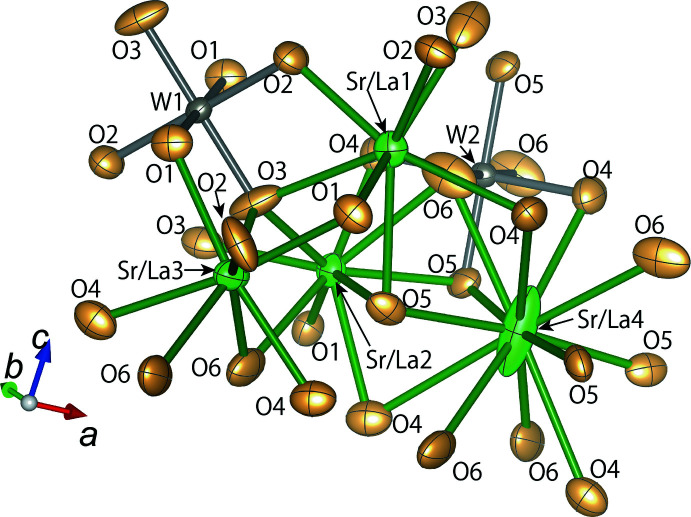
The principal building units in the crystal structure of Sr_9_La_2_(WO_6_)_4_ with displacement ellipsoids drawn at the 99% probability level. Symmetry codes refer to Table 1 ▸.

The unit-cell parameters of Sr_9_La_2_(WO_6_)_4_ determined in the present investigation are consistent with those reported in previous studies (Smirnov *et al.*, 1987 ▸; Kemmler-Sack & Ehmann, 1981 ▸). Fig. 1 ▸ displays the principal building units in the crystal structure of Sr_9_La_2_(WO_6_)_4_. W1 (multiplicity and Wyckoff letter 8*d* with site symmetry 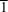 ) and W2 (8*c*, 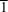 ) each are located at the center of a [WO_6_] octa­hedron. The [WO_6_] octa­hedra are isolated and surrounded by mixed-occupied (Sr,La) atoms. As detailed in Table 1 ▸, the inter­atomic distances between W and O are 1.901 (4)–1.934 (4) Å (average: 1.922 Å) for W1—O and 1.891 (4)–1.967 (4) Å (average: 1.925 Å) for W2—O. The bond-valence sums (BVS; Brown & Altermatt, 1985 ▸) for W1 and W2, as calculated using the parameters for W—O (*R*_0_ = 1.921, *B* = 0.37) (Brese & O’Keeffe, 1991 ▸), are 5.994 and 5.957 valence units, respectively. These values are consistent with the valence state +VI for W.

The Sr/La occupancies for (Sr/La)1 (16*f*, 1), (Sr/La)2 (16*f*, 1), (Sr/La)3 (8*e*, 2..), and (Sr/La)4 (4*a*, 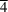 ..) are 0.6384/0.3616 (19), 0.8913/0.1087 (18), 0.948/0.052 (4), and 0.985/0.015 (7), respectively. The inter­atomic distances between (Sr/La) and O and the coordination numbers of the cations are 2.333 (4)–2.861 (4) Å (average: 2.611 Å) and 8 for (Sr/La)1—O; 2.470 (4)–2.877 (5) Å (average: 2.660 Å) and 8 for (Sr/La)2—O; 2.557 (4)–3.220 (4) Å (average: 2.761 Å) and 10 for (Sr/La)3—O; and 2.607 (4)–3.131 (4) Å (average: 2.912 Å) and 12 for (Sr/La)4—O. As the La occupancy increases, the (Sr/La)—O inter­atomic distance decreases.

**Figure 2:**
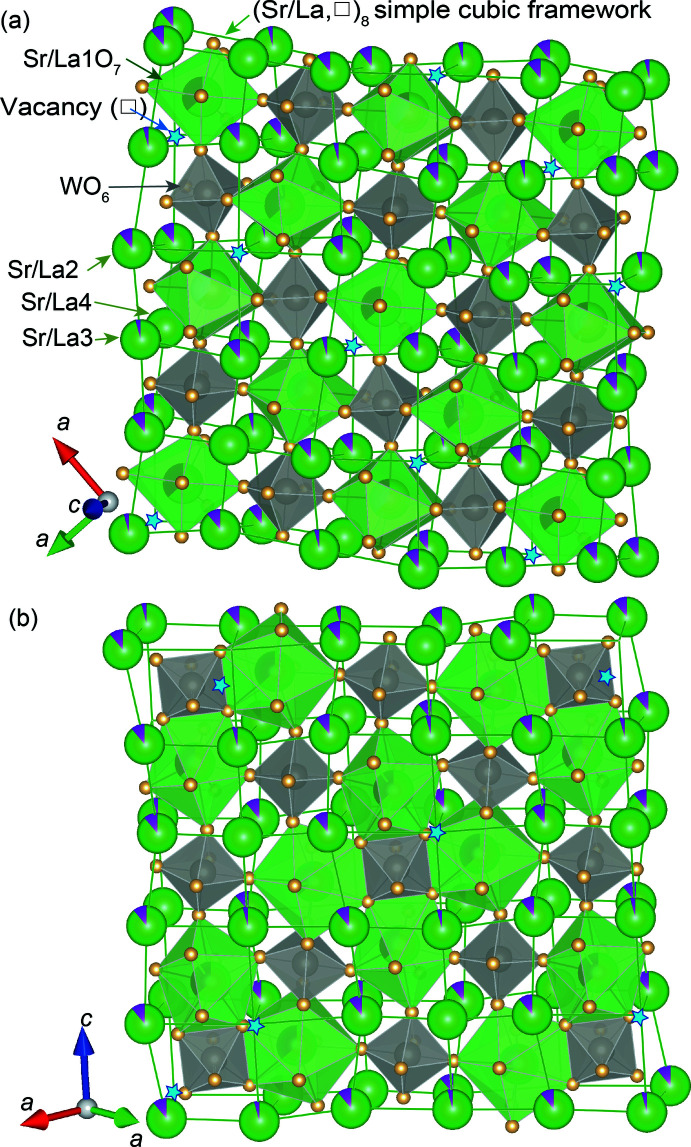
[WO_6_] octa­hedra and [(Sr/La)1O_8_] polyhedra alternately distributed in the distorted (Sr/La2–4,□)_8_ lattice as illustrated for the planes parallel to (001) in (*a*) and (110) in (*b*). Note that [WO_6_] octa­hedra and [(Sr/La)1O_8_] polyhedra are connected to each other by vertex- or edge-sharing.

The crystal structures of alkaline-earth and rare-earth tungstates are often described in relation to the double-perovskite structure type (Kemmler-Sack & Ehmann, 1981 ▸; Betz *et al.*, 1982 ▸; Blasse & Kemmler-Sack, 1983 ▸; King *et al.*, 2010 ▸; Ijdo *et al.*, 2016 ▸). In the double-perovskite (*A*_2_ *BB*′O_6_) structure, *B* and *B*′ atoms alternately occupy the *B* site of the perovskite (*AB*O_3_) structure. The *B* site is at the center of an octa­hedron formed by O atoms, and the vertex-sharing [*B*O_6_] and [*B*′O_6_] octa­hedra regularly align in the *A*_8_ simple cubic lattice frame in the double-perovskite structure. In case of the structure of Sr_9_La_2_(WO_6_)_4_, a (Sr/La,□)_8_ distorted simple lattice can be derived by connecting the Sr-rich sites of (Sr/La)2, (Sr/La)3, and (Sr/La)4 and a vacancy site at (1/2, 3/4, 1/8), as shown in Fig. 2 ▸. In the distorted lattice, the [WO_6_] octa­hedra and the [(Sr/La)1O_8_] polyhedra are alternately located by sharing four vertices and two edges of the [(Sr/La)1O_8_] polyhedra (Fig. 2 ▸).

The crystal structure of Sr_9_La_2_(WO_6_)_4_ is isotypic with those of Sr_11_(ReO_6_)_4_ [*a* = 11.6779 (1), *c* = 16.1488 (2); Bramnik *et al.*, 2000 ▸], Ba_11_(OsO_6_)_4_ [*a* = 12.2414 (1), *c* = 16.6685 (1); Wakeshima & Hinatsu, 2005 ▸], La_9_Sr(IrO_6_)_4_ [*a* = 11.5955 (11), *c* = 16.2531 (15); Ferreira *et al.*, 2018 ▸], and Sr_11_(MoO_6_)_4_ [*a* = 11.6107 (6), *c* = 16.4219 (13); Löpez *et al.*, 2016 ▸].

## 3. Synthesis and crystallization

Raw powdered materials of SrCO_3_ (Hakushin Chemical Laboratory, 98%), WO_3_ (Furuuchi Chemical, 99.99%), and La_2_O_3_ (FUJIFILM Wako Pure Chemical, 99.99%; calcined at 1273 K in advance) were weighed in a Sr:La:W molar ratio of 9:2:4, mixed in an agate mortar, and pressed into a cylindrical pellet with a diameter of 6 mm. The pellet was placed on a Pt plate in an alumina crucible with a lid (Nikkato, SSA-S) and heated to 1473 K at a rate of 300 K h^−1^ in a furnace. This temperature was maintained for 10 h, and the power to the heater of the furnace was then shut off. After the sample had cooled to room temperature, the sintered pellet was crushed, pressed into a pellet, and heated again under the same conditions. This procedure was performed three times. Part of the sintered pellet was then placed on a Pt plate in an alumina crucible, heated at 1673 K for 6 h, and cooled to room temperature at a rate of −400 K h^−1^. The obtained crystalline sample was an aggregate consisting of ∼50 µm single-crystalline grains. A single crystal selected from the aggregate was placed on top of a glass fiber for X-ray structure analysis. Another single crystal was embedded in resin, mirror polished, and carbon coated in preparation for chemical analysis using an electron microprobe analyzer (EPMA; JEOL JXA-8200). The chemical composition determined by EPMA was Sr: 23.2 (4), La: 4.8 (1), W: 10.3 (3), and O: 61.7 (5) wt%. The Sr:La:W:O atomic ratio of 9.1 (1): 1.9 (1): 4.0 (1): 24.0 (2) calculated from the composition is consistent with the chemical formula Sr_9_La_2_(WO_6_)_4_.

## 4. Refinement

The results of the crystal structure analysis are summarized in Table 2 ▸. An initial structure model with two W sites, four Sr sites, and six O sites using isotropic displacement parameters showed residual electron density distribution around the four Sr sites. These sites were changed to Sr/La mixed sites, and their occupancies were refined under consideration of full occupancy, resulting in an Sr:La:W:O atomic ratio of 35.6:8.4:16:96. Given the charge balance, the numbers of Sr and La atoms in the unit cell was constrained to be 36 and 8, respectively.

## Supplementary Material

Crystal structure: contains datablock(s) I. DOI: 10.1107/S2056989022006648/wm5650sup1.cif

Structure factors: contains datablock(s) I. DOI: 10.1107/S2056989022006648/wm5650Isup2.hkl

CCDC reference: 2182445

Additional supporting information:  crystallographic information; 3D view; checkCIF report

## Figures and Tables

**Table 1:** Selected bond lengths (Å)

Sr1/La1—O6^i^	2.333 (4)	Sr3/La3—O1^x^	3.220 (4)
Sr1/La1—O2	2.438 (4)	Sr4/La4—O1	2.607 (4)
Sr1/La1—O2^ii^	2.453 (4)	Sr4/La4—O1^vi^	2.607 (4)
Sr1/La1—O4^iii^	2.458 (4)	Sr4/La4—O1^xi^	2.607 (4)
Sr1/La1—O5^iv^	2.728 (4)	Sr4/La4—O1^ix^	2.607 (4)
Sr1/La1—O3^iii^	2.765 (5)	Sr4/La4—O4	2.998 (5)
Sr1/La1—O3	2.849 (5)	Sr4/La4—O4^xi^	2.998 (5)
Sr1/La1—O1^iii^	2.861 (4)	Sr4/La4—O4^ix^	2.998 (5)
Sr2/La2—O3^v^	2.470 (4)	Sr4/La4—O4^vi^	2.998 (5)
Sr2/La2—O1^vi^	2.548 (4)	Sr4/La4—O5^i^	3.131 (4)
Sr2/La2—O6	2.599 (4)	Sr4/La4—O5^xii^	3.131 (4)
Sr2/La2—O2^vii^	2.603 (4)	Sr4/La4—O5^v^	3.131 (4)
Sr2/La2—O1	2.642 (4)	Sr4/La4—O5^viii^	3.131 (4)
Sr2/La2—O5	2.652 (4)	W1—O3	1.901 (4)
Sr2/La2—O5^v^	2.704 (4)	W1—O3^xiii^	1.901 (4)
Sr2/La2—O4	2.777 (4)	W1—O6^viii^	1.930 (4)
Sr2/La2—O4^v^	2.877 (5)	W1—O6^xiv^	1.930 (4)
Sr3/La3—O6^i^	2.557 (4)	W1—O2	1.934 (4)
Sr3/La3—O6^viii^	2.557 (4)	W1—O2^xiii^	1.934 (4)
Sr3/La3—O5^viii^	2.596 (4)	W2—O4^xi^	1.891 (4)
Sr3/La3—O5^i^	2.596 (4)	W2—O4^v^	1.891 (4)
Sr3/La3—O3	2.660 (4)	W2—O1^xv^	1.917 (4)
Sr3/La3—O3^ix^	2.660 (4)	W2—O1	1.917 (4)
Sr3/La3—O4^ix^	2.773 (4)	W2—O5^xi^	1.967 (4)
Sr3/La3—O4	2.773 (4)	W2—O5^v^	1.967 (4)
Sr3/La3—O1^iii^	3.220 (4)		

Symmetry codes: (i) 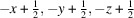 ; (ii) 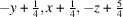 ; (iii) 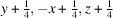 ; (iv) 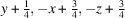 ; (v) 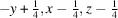 ; (vi) 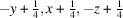 ; (vii) 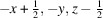 ; (viii) 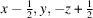 ; (ix) 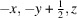 ; (x) 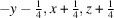 ; (xi) 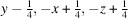 ; (xii) 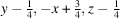 ; (xiii) 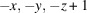 ; (xiv) 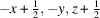 ; (xv) 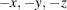 .

**Table 2:** Experimental details

Crystal data	
Chemical formula	Sr_9_La_2_(WO_6_)_4_
*M*_r_	2185.80
Crystal system, space group	Tetragonal, *I*4_1_/*a*
Temperature (K)	300
*a*, *c* (Å)	11.6365 (3), 16.3040 (4)
*V* (Å^3^)	2207.69 (13)
*Z*	4
Radiation type	Mo *K*α
μ (mm^−1^)	46.16
Crystal size (mm)	0.05 × 0.04 × 0.03

Data collection	
Diffractometer	Bruker D8 QUEST
Absorption correction	Multi-scan (*SADABS*; Krause *et al.*, 2015 ▸)
*T*_min_, *T*_max_	0.20, 0.33
No. of measured, independent and observed [*I* > 2σ(*I*)] reflections	62981, 2106, 1972
*R*_int_	0.048
(sin θ/λ)_max_ (Å^−1^)	0.770

Refinement	
*R*[*F* ^2^ > 2σ(*F* ^2^)], *wR*(*F* ^2^), *S*	0.025, 0.046, 1.37
No. of reflections	2106
No. of parameters	97
No. of restraints	1
Δρ_max_, Δρ_min_ (e Å^−3^)	1.14, −1.50

Computer programs: *APEX3* and *SAINT* (Bruker, 2018 ▸), *SHELXT2014/5* (Sheldrick, 2015*a* ▸), *SHELXL2018/3* (Sheldrick, 2015*b* ▸), *VESTA* (Momma & Izumi, 2011 ▸) and *publCIF* (Westrip, 2010 ▸).

## supplementary crystallographic information

### Crystal data

**Table:**

Sr_9_La_2_(WO_6_)_4_	*D*_x_ = 6.576 Mg m^−^^3^
*M**_r_* = 2185.80	Mo *K*α radiation, λ = 0.71073 Å
Tetragonal, *I*4_1_/*a*	Cell parameters from 9792 reflections
*a* = 11.6365 (3) Å	θ = 3.5–33.2°
*c* = 16.3040 (4) Å	µ = 46.16 mm^−^^1^
*V* = 2207.69 (13) Å^3^	*T* = 300 K
*Z* = 4	Granular, translucent colourless
*F*(000) = 3776	0.05 × 0.04 × 0.03 mm

### Data collection

**Table:**

Bruker D8 QUEST diffractometer	2106 independent reflections
Radiation source: sealed X-ray tube	1972 reflections with *I* > 2σ(*I*)
Detector resolution: 7.3910 pixels mm^-1^	*R*_int_ = 0.048
ω and σcans	θ_max_ = 33.2°, θ_min_ = 2.2°
Absorption correction: multi-scan (*SADABS*; Krause *et al.*, 2015)	*h* = −17→17
*T*_min_ = 0.20, *T*_max_ = 0.33	*k* = −17→17
62981 measured reflections	*l* = −25→25

### Refinement

**Table:**

Refinement on *F*^2^	1 restraint
Least-squares matrix: full	*w* = 1/[σ^2^(*F*_o_^2^) + 62.4087*P*] where *P* = (*F*_o_^2^ + 2*F*_c_^2^)/3
*R*[*F*^2^ > 2σ(*F*^2^)] = 0.025	(Δ/σ)_max_ = 0.001
*wR*(*F*^2^) = 0.046	Δρ_max_ = 1.14 e Å^−^^3^
*S* = 1.37	Δρ_min_ = −1.50 e Å^−^^3^
2106 reflections	Extinction correction: SHELXL-2014/7 (Sheldrick, 2015b), Fc^*^=kFc[1+0.001xFc^2^λ^3^/sin(2θ)]^-1/4^
97 parameters	Extinction coefficient: 0.000055 (5)

### Special details

**Table:**

Geometry. All esds (except the esd in the dihedral angle between two l.s. planes) are estimated using the full covariance matrix. The cell esds are taken into account individually in the estimation of esds in distances, angles and torsion angles; correlations between esds in cell parameters are only used when they are defined by crystal symmetry. An approximate (isotropic) treatment of cell esds is used for estimating esds involving l.s. planes.

### Fractional atomic coordinates and isotropic or equivalent isotropic displacement parameters (Å^2^)

**Table:**

	*x*	*y*	*z*	*U*_iso_*/*U*_eq_	Occ. (<1)
Sr1	0.20878 (3)	0.22538 (3)	0.53417 (2)	0.00769 (8)	0.6384 (19)
La1	0.20878 (3)	0.22538 (3)	0.53417 (2)	0.00769 (8)	0.3616 (19)
Sr2	0.23647 (4)	0.04341 (4)	0.11357 (3)	0.00718 (9)	0.8913 (18)
La2	0.23647 (4)	0.04341 (4)	0.11357 (3)	0.00718 (9)	0.1087 (18)
Sr3	0.0000	0.2500	0.36535 (4)	0.00934 (14)	0.948 (4)
La3	0.0000	0.2500	0.36535 (4)	0.00934 (14)	0.052 (4)
Sr4	0.0000	0.2500	0.1250	0.0267 (4)	0.985 (7)
La4	0.0000	0.2500	0.1250	0.0267 (4)	0.015 (7)
W1	0.0000	0.0000	0.5000	0.00522 (6)	
W2	0.0000	0.0000	0.0000	0.00502 (6)	
O1	0.0101 (3)	0.0266 (3)	0.1158 (2)	0.0093 (7)	
O2	0.0795 (3)	0.0786 (3)	0.5877 (2)	0.0099 (7)	
O3	0.1059 (4)	0.0651 (4)	0.4243 (3)	0.0137 (8)	
O4	0.1383 (4)	0.1321 (4)	0.2554 (3)	0.0148 (8)	
O5	0.3675 (3)	0.1315 (3)	0.2308 (2)	0.0109 (7)	
O6	0.4011 (3)	0.1285 (3)	0.0246 (2)	0.0096 (7)	

### Atomic displacement parameters (Å^2^)

**Table:**

	*U*^11^	*U*^22^	*U*^33^	*U*^12^	*U*^13^	*U*^23^
Sr1	0.00728 (15)	0.00640 (15)	0.00939 (16)	0.00166 (12)	−0.00049 (12)	0.00060 (12)
La1	0.00728 (15)	0.00640 (15)	0.00939 (16)	0.00166 (12)	−0.00049 (12)	0.00060 (12)
Sr2	0.00722 (17)	0.00602 (17)	0.00830 (18)	−0.00036 (14)	0.00015 (14)	−0.00025 (14)
La2	0.00722 (17)	0.00602 (17)	0.00830 (18)	−0.00036 (14)	0.00015 (14)	−0.00025 (14)
Sr3	0.0127 (3)	0.0080 (3)	0.0074 (3)	−0.0024 (2)	0.000	0.000
La3	0.0127 (3)	0.0080 (3)	0.0074 (3)	−0.0024 (2)	0.000	0.000
Sr4	0.0091 (3)	0.0091 (3)	0.0620 (10)	0.000	0.000	0.000
La4	0.0091 (3)	0.0091 (3)	0.0620 (10)	0.000	0.000	0.000
W1	0.00463 (11)	0.00540 (11)	0.00562 (11)	−0.00008 (8)	0.00033 (8)	0.00085 (8)
W2	0.00472 (11)	0.00554 (11)	0.00480 (11)	0.00064 (8)	−0.00001 (8)	−0.00041 (8)
O1	0.0062 (14)	0.0135 (17)	0.0081 (16)	−0.0004 (12)	−0.0012 (12)	−0.0019 (13)
O2	0.0116 (16)	0.0106 (16)	0.0073 (16)	−0.0022 (13)	0.0000 (13)	−0.0007 (13)
O3	0.0128 (17)	0.0134 (18)	0.0149 (19)	0.0023 (14)	0.0071 (14)	0.0062 (14)
O4	0.0184 (19)	0.0141 (18)	0.0118 (18)	−0.0098 (15)	0.0025 (15)	−0.0006 (15)
O5	0.0117 (17)	0.0112 (17)	0.0098 (17)	0.0032 (13)	0.0018 (13)	0.0006 (13)
O6	0.0097 (16)	0.0092 (16)	0.0098 (16)	0.0027 (12)	−0.0009 (13)	−0.0005 (13)

### Geometric parameters (Å, º)

**Table:**

Sr1/La1—O6^i^	2.333 (4)	Sr3/La3—O1^xi^	3.220 (4)
Sr1/La1—O2	2.438 (4)	Sr3/La3—W2^xi^	3.4641 (4)
Sr1/La1—O2^ii^	2.453 (4)	Sr3/La3—W2^iii^	3.4641 (4)
Sr1/La1—O4^iii^	2.458 (4)	Sr4/La4—O1	2.607 (4)
Sr1/La1—O5^iv^	2.728 (4)	Sr4/La4—O1^vii^	2.607 (4)
Sr1/La1—O3^iii^	2.765 (5)	Sr4/La4—O1^xii^	2.607 (4)
Sr1/La1—O3	2.849 (5)	Sr4/La4—O1^x^	2.607 (4)
Sr1/La1—O1^iii^	2.861 (4)	Sr4/La4—O4	2.998 (5)
Sr1/La1—Sr2/La2^v^	3.4446 (6)	Sr4/La4—O4^xii^	2.998 (5)
Sr1/La1—Sr2/La2^v^	3.4446 (6)	Sr4/La4—O4^x^	2.998 (5)
Sr1/La1—W1^iii^	3.5630 (4)	Sr4/La4—O4^vii^	2.998 (5)
Sr1/La1—W1	3.6181 (4)	Sr4/La4—O5^i^	3.131 (4)
Sr2/La2—O3^vi^	2.470 (4)	Sr4/La4—O5^xiii^	3.131 (4)
Sr2/La2—O1^vii^	2.548 (4)	Sr4/La4—O5^vi^	3.131 (4)
Sr2/La2—O6	2.599 (4)	Sr4/La4—O5^ix^	3.131 (4)
Sr2/La2—O2^viii^	2.603 (4)	W1—O3	1.901 (4)
Sr2/La2—O1	2.642 (4)	W1—O3^xiv^	1.901 (4)
Sr2/La2—O5	2.652 (4)	W1—O6^ix^	1.930 (4)
Sr2/La2—O5^vi^	2.704 (4)	W1—O6^v^	1.930 (4)
Sr2/La2—O4	2.777 (4)	W1—O2	1.934 (4)
Sr2/La2—O4^vi^	2.877 (5)	W1—O2^xiv^	1.934 (4)
Sr2/La2—W2^iii^	3.2790 (4)	W1—Sr1/La1^xv^	3.5629 (4)
Sr2/La2—W2	3.3549 (4)	W1—Sr1/La1^vi^	3.5629 (4)
Sr2/La2—Sr1^viii^	3.4446 (6)	W1—Sr2/La2^ix^	3.6177 (4)
Sr3/La3—O6^i^	2.557 (4)	W1—Sr2/La2^v^	3.6177 (4)
Sr3/La3—O6^ix^	2.557 (4)	W2—O4^xii^	1.891 (4)
Sr3/La3—O5^ix^	2.596 (4)	W2—O4^vi^	1.891 (4)
Sr3/La3—O5^i^	2.596 (4)	W2—O1^xvi^	1.917 (4)
Sr3/La3—O3	2.660 (4)	W2—O1	1.917 (4)
Sr3/La3—O3^x^	2.660 (4)	W2—O5^xii^	1.967 (4)
Sr3/La3—O4^x^	2.773 (4)	W2—O5^vi^	1.967 (4)
Sr3/La3—O4	2.773 (4)	W2—Sr2/La2^xii^	3.2790 (4)
Sr3/La3—O1^iii^	3.220 (4)	W2—Sr2/La2^vi^	3.2790 (4)
			
O6^i^—Sr1/La1—O2	108.65 (13)	W2^xi^—Sr3/La3—W2^iii^	114.236 (19)
O6^i^—Sr1/La1—O2^ii^	83.84 (13)	O1—Sr4/La4—O1^vii^	90.189 (10)
O2—Sr1/La1—O2^ii^	86.10 (14)	O1—Sr4/La4—O1^xii^	90.189 (10)
O6^i^—Sr1/La1—O4^iii^	139.77 (14)	O1^vii^—Sr4/La4—O1^xii^	173.41 (17)
O2—Sr1/La1—O4^iii^	101.40 (14)	O1—Sr4/La4—O1^x^	173.41 (17)
O2^ii^—Sr1/La1—O4^iii^	125.03 (13)	O1^vii^—Sr4/La4—O1^x^	90.189 (10)
O6^i^—Sr1/La1—O5^iv^	83.02 (13)	O1^xii^—Sr4/La4—O1^x^	90.189 (10)
O2—Sr1/La1—O5^iv^	163.00 (12)	O1—Sr4/La4—O4	63.92 (11)
O2^ii^—Sr1/La1—O5^iv^	82.86 (13)	O1^vii^—Sr4/La4—O4	56.25 (11)
O4^iii^—Sr1/La1—O5^iv^	74.87 (14)	O1^xii^—Sr4/La4—O4	118.30 (11)
O6^i^—Sr1/La1—O3^iii^	146.51 (13)	O1^x^—Sr4/La4—O4	121.40 (11)
O2—Sr1/La1—O3^iii^	74.94 (12)	O1—Sr4/La4—O4^xii^	56.25 (11)
O2^ii^—Sr1/La1—O3^iii^	62.93 (12)	O1^vii^—Sr4/La4—O4^xii^	121.40 (11)
O4^iii^—Sr1/La1—O3^iii^	66.83 (13)	O1^xii^—Sr4/La4—O4^xii^	63.92 (11)
O5^iv^—Sr1/La1—O3^iii^	88.51 (12)	O1^x^—Sr4/La4—O4^xii^	118.30 (11)
O6^i^—Sr1/La1—O3	89.34 (13)	O4—Sr4/La4—O4^xii^	120.17 (10)
O2—Sr1/La1—O3	60.49 (12)	O1—Sr4/La4—O4^x^	121.40 (11)
O2^ii^—Sr1/La1—O3	141.63 (12)	O1^vii^—Sr4/La4—O4^x^	118.30 (11)
O4^iii^—Sr1/La1—O3	82.66 (13)	O1^xii^—Sr4/La4—O4^x^	56.25 (11)
O5^iv^—Sr1/La1—O3	133.77 (12)	O1^x^—Sr4/La4—O4^x^	63.92 (11)
O3^iii^—Sr1/La1—O3	118.90 (13)	O4—Sr4/La4—O4^x^	89.71 (17)
O6^i^—Sr1/La1—O1^iii^	73.37 (12)	O4^xii^—Sr4/La4—O4^x^	120.17 (10)
O2—Sr1/La1—O1^iii^	123.73 (12)	O1—Sr4/La4—O4^vii^	118.30 (11)
O2^ii^—Sr1/La1—O1^iii^	146.75 (12)	O1^vii^—Sr4/La4—O4^vii^	63.92 (11)
O4^iii^—Sr1/La1—O1^iii^	67.80 (12)	O1^xii^—Sr4/La4—O4^vii^	121.40 (11)
O5^iv^—Sr1/La1—O1^iii^	70.80 (11)	O1^x^—Sr4/La4—O4^vii^	56.25 (11)
O3^iii^—Sr1/La1—O1^iii^	133.60 (11)	O4—Sr4/La4—O4^vii^	120.17 (10)
O3—Sr1/La1—O1^iii^	63.35 (11)	O4^xii^—Sr4/La4—O4^vii^	89.71 (17)
Sr2/La2^v^—Sr1/La1—W1	61.565 (10)	O4^x^—Sr4/La4—O4^vii^	120.17 (10)
W1^iii^—Sr1/La1—W1	107.502 (10)	O1—Sr4/La4—O5^i^	117.41 (11)
O3^vi^—Sr2/La2—O1^vii^	143.76 (14)	O1^vii^—Sr4/La4—O5^i^	56.35 (11)
O3^vi^—Sr2/La2—O6	137.69 (13)	O1^xii^—Sr4/La4—O5^i^	117.90 (11)
O1^vii^—Sr2/La2—O6	74.95 (12)	O1^x^—Sr4/La4—O5^i^	68.03 (11)
O3^vi^—Sr2/La2—O2^viii^	77.47 (13)	O4—Sr4/La4—O5^i^	53.49 (10)
O1^vii^—Sr2/La2—O2^viii^	127.58 (12)	O4^xii^—Sr4/La4—O5^i^	173.65 (10)
O6—Sr2/La2—O2^viii^	60.75 (12)	O4^x^—Sr4/La4—O5^i^	62.02 (10)
O3^vi^—Sr2/La2—O1	71.74 (13)	O4^vii^—Sr4/La4—O5^i^	94.03 (11)
O1^vii^—Sr2/La2—O1	90.70 (17)	O1—Sr4/La4—O5^xiii^	117.90 (11)
O6—Sr2/La2—O1	140.44 (12)	O1^vii^—Sr4/La4—O5^xiii^	117.41 (11)
O2^viii^—Sr2/La2—O1	141.44 (12)	O1^xii^—Sr4/La4—O5^xiii^	68.03 (11)
O3^vi^—Sr2/La2—O5	101.06 (13)	O1^x^—Sr4/La4—O5^xiii^	56.35 (11)
O1^vii^—Sr2/La2—O5	63.69 (12)	O4—Sr4/La4—O5^xiii^	173.65 (10)
O6—Sr2/La2—O5	80.29 (12)	O4^xii^—Sr4/La4—O5^xiii^	62.02 (10)
O2^viii^—Sr2/La2—O5	81.63 (12)	O4^x^—Sr4/La4—O5^xiii^	94.03 (11)
O1—Sr2/La2—O5	126.30 (12)	O4^vii^—Sr4/La4—O5^xiii^	53.49 (10)
O3^vi^—Sr2/La2—O5^vi^	118.64 (12)	O5^i^—Sr4/La4—O5^xiii^	124.29 (9)
O1^vii^—Sr2/La2—O5^vi^	76.11 (12)	O1—Sr4/La4—O5^vi^	56.35 (11)
O6—Sr2/La2—O5^vi^	78.82 (12)	O1^vii^—Sr4/La4—O5^vi^	68.03 (11)
O2^viii^—Sr2/La2—O5^vi^	117.51 (12)	O1^xii^—Sr4/La4—O5^vi^	117.41 (11)
O1—Sr2/La2—O5^vi^	61.87 (12)	O1^x^—Sr4/La4—O5^vi^	117.90 (11)
O5—Sr2/La2—O5^vi^	138.23 (9)	O4—Sr4/La4—O5^vi^	94.03 (11)
O3^vi^—Sr2/La2—O4	83.96 (13)	O4^xii^—Sr4/La4—O5^vi^	53.49 (10)
O1^vii^—Sr2/La2—O4	59.86 (12)	O4^x^—Sr4/La4—O5^vi^	173.65 (10)
O6—Sr2/La2—O4	128.74 (12)	O4^vii^—Sr4/La4—O5^vi^	62.02 (10)
O2^viii^—Sr2/La2—O4	132.50 (13)	O5^i^—Sr4/La4—O5^vi^	124.29 (9)
O1—Sr2/La2—O4	66.80 (12)	O5^xiii^—Sr4/La4—O5^vi^	82.71 (14)
O5—Sr2/La2—O4	59.51 (12)	O1—Sr4/La4—O5^ix^	68.03 (11)
O5^vi^—Sr2/La2—O4	109.81 (13)	O1^vii^—Sr4/La4—O5^ix^	117.90 (11)
O3^vi^—Sr2/La2—O4^vi^	64.87 (13)	O1^xii^—Sr4/La4—O5^ix^	56.35 (11)
O1^vii^—Sr2/La2—O4^vi^	132.31 (11)	O1^x^—Sr4/La4—O5^ix^	117.41 (11)
O6—Sr2/La2—O4^vi^	104.51 (12)	O4—Sr4/La4—O5^ix^	62.02 (10)
O2^viii^—Sr2/La2—O4^vi^	87.33 (12)	O4^xii^—Sr4/La4—O5^ix^	94.03 (11)
O1—Sr2/La2—O4^vi^	58.90 (11)	O4^x^—Sr4/La4—O5^ix^	53.49 (10)
O5—Sr2/La2—O4^vi^	163.87 (12)	O4^vii^—Sr4/La4—O5^ix^	173.65 (10)
O5^vi^—Sr2/La2—O4^vi^	57.69 (11)	O5^i^—Sr4/La4—O5^ix^	82.71 (14)
O4—Sr2/La2—O4^vi^	123.12 (9)	O5^xiii^—Sr4/La4—O5^ix^	124.29 (9)
W2^iii^—Sr2/La2—W2	121.613 (13)	O5^vi^—Sr4/La4—O5^ix^	124.29 (9)
W2^iii^—Sr2/La2—Sr1/La1^viii^	155.754 (16)	O3—W1—O3^xiv^	180.0
W2—Sr2/La2—Sr1/La1^viii^	78.905 (11)	O3—W1—O6^ix^	86.73 (17)
O6^i^—Sr3/La3—O6^ix^	90.91 (18)	O3^xiv^—W1—O6^ix^	93.27 (17)
O6^i^—Sr3/La3—O5^ix^	169.96 (12)	O3—W1—O6^v^	93.27 (17)
O6^ix^—Sr3/La3—O5^ix^	82.12 (12)	O3^xiv^—W1—O6^v^	86.73 (17)
O6^i^—Sr3/La3—O5^i^	82.12 (12)	O6^ix^—W1—O6^v^	180.0 (2)
O6^ix^—Sr3/La3—O5^i^	169.96 (12)	O3—W1—O2	88.94 (18)
O5^ix^—Sr3/La3—O5^i^	105.66 (18)	O3^xiv^—W1—O2	91.06 (18)
O6^i^—Sr3/La3—O3	89.14 (13)	O6^ix^—W1—O2	94.18 (16)
O6^ix^—Sr3/La3—O3	60.52 (12)	O6^v^—W1—O2	85.82 (16)
O5^ix^—Sr3/La3—O3	93.66 (13)	O3—W1—O2^xiv^	91.07 (18)
O5^i^—Sr3/La3—O3	111.89 (12)	O3^xiv^—W1—O2^xiv^	88.93 (18)
O6^i^—Sr3/La3—O3^x^	60.52 (12)	O6^ix^—W1—O2^xiv^	85.82 (16)
O6^ix^—Sr3/La3—O3^x^	89.14 (13)	O6^v^—W1—O2^xiv^	94.18 (16)
O5^ix^—Sr3/La3—O3^x^	111.89 (12)	O2—W1—O2^xiv^	180.0
O5^i^—Sr3/La3—O3^x^	93.66 (13)	Sr1/La1^xv^—W1—Sr1/La1^vi^	180.0
O3—Sr3/La3—O3^x^	137.62 (19)	Sr1/La1^vi^—W1—Sr1/La1^vi^	0.0
O6^i^—Sr3/La3—O4^x^	116.21 (12)	Sr1/La1^xv^—W1—Sr1/La1^xv^	0.0
O6^ix^—Sr3/La3—O4^x^	117.76 (12)	Sr1/La1^vi^—W1—Sr1/La1^xv^	180.0
O5^ix^—Sr3/La3—O4^x^	61.78 (12)	Sr1/La1^xv^—W1—Sr2/La2^ix^	114.745 (9)
O5^i^—Sr3/La3—O4^x^	72.02 (12)	Sr1/La1^vi^—W1—Sr2/La2^ix^	65.255 (9)
O3—Sr3/La3—O4^x^	154.56 (13)	Sr1/La1^xv^—W1—Sr2/La2^ix^	114.745 (9)
O3^x^—Sr3/La3—O4^x^	64.19 (13)	Sr1/La1^xv^—W1—Sr2/La2^v^	65.255 (9)
O6^i^—Sr3/La3—O4	117.76 (12)	Sr1/La1^vi^—W1—Sr2/La2^v^	114.745 (9)
O6^ix^—Sr3/La3—O4	116.21 (12)	Sr2/La2^ix^—W1—Sr2/La2^v^	180.000 (12)
O5^ix^—Sr3/La3—O4	72.02 (12)	O4^xii^—W2—O4^vi^	180.0 (3)
O5^i^—Sr3/La3—O4	61.78 (12)	O4^xii^—W2—O1^xvi^	91.21 (17)
O3—Sr3/La3—O4	64.19 (13)	O4^vi^—W2—O1^xvi^	88.79 (17)
O3^x^—Sr3/La3—O4	154.56 (13)	O4^xii^—W2—O1	88.80 (17)
O4^x^—Sr3/La3—O4	99.40 (19)	O4^vi^—W2—O1	91.20 (17)
O6^i^—Sr3/La3—O1^iii^	64.45 (11)	O1^xvi^—W2—O1	180.0
O6^ix^—Sr3/La3—O1^iii^	115.34 (11)	O4^xii^—W2—O5^xii^	88.65 (18)
O5^ix^—Sr3/La3—O1^iii^	125.14 (11)	O4^vi^—W2—O5^xii^	91.35 (18)
O5^i^—Sr3/La3—O1^iii^	55.05 (11)	O1^xvi^—W2—O5^xii^	90.08 (16)
O3—Sr3/La3—O1^iii^	60.43 (11)	O1—W2—O5^xii^	89.92 (16)
O3^x^—Sr3/La3—O1^iii^	119.46 (11)	O4^xii^—W2—O5^vi^	91.35 (18)
O4^x^—Sr3/La3—O1^iii^	126.84 (11)	O4^vi^—W2—O5^vi^	88.65 (18)
O4—Sr3/La3—O1^iii^	53.37 (11)	O1^xvi^—W2—O5^vi^	89.92 (16)
O6^i^—Sr3/La3—O1^xi^	115.34 (11)	O1—W2—O5^vi^	90.08 (16)
O6^ix^—Sr3/La3—O1^xi^	64.45 (11)	O5^xii^—W2—O5^vi^	180.0 (3)
O5^ix^—Sr3/La3—O1^xi^	55.05 (11)	Sr2/La2^xii^—W2—Sr2/La2^vi^	180.0
O5^i^—Sr3/La3—O1^xi^	125.14 (11)	Sr2/La2^xii^—W2—Sr2/La2^xii^	0.0
O3—Sr3/La3—O1^xi^	119.46 (11)	Sr2/La2^vi^—W2—Sr2/La2^xii^	180.00 (2)
O3^x^—Sr3/La3—O1^xi^	60.43 (11)	Sr2/La2^vi^—W2—Sr2/La2^vi^	0.000 (11)
O4^x^—Sr3/La3—O1^xi^	53.37 (11)	Sr2/La2^xii^—W2—Sr2/La2	102.7
O4—Sr3/La3—O1^xi^	126.84 (11)	Sr2/La2^vi^—W2—Sr2/La2	77.309 (8)
O1^iii^—Sr3/La3—O1^xi^	179.73 (14)	Sr2/La2^xii^—W2—Sr2/La2	102.691 (8)

Symmetry codes: (i) −*x*+1/2, −*y*+1/2, −*z*+1/2; (ii) −*y*+1/4, *x*+1/4, −*z*+5/4; (iii) *y*+1/4, −*x*+1/4, *z*+1/4; (iv) *y*+1/4, −*x*+3/4, −*z*+3/4; (v) −*x*+1/2, −*y*, *z*+1/2; (vi) −*y*+1/4, *x*−1/4, *z*−1/4; (vii) −*y*+1/4, *x*+1/4, −*z*+1/4; (viii) −*x*+1/2, −*y*, *z*−1/2; (ix) *x*−1/2, *y*, −*z*+1/2; (x) −*x*, −*y*+1/2, *z*; (xi) −*y*−1/4, *x*+1/4, *z*+1/4; (xii) *y*−1/4, −*x*+1/4, −*z*+1/4; (xiii) *y*−1/4, −*x*+3/4, *z*−1/4; (xiv) −*x*, −*y*, −*z*+1; (xv) *y*−1/4, −*x*+1/4, −*z*+5/4; (xvi) −*x*, −*y*, −*z*.
